# Effects of metformin and Exenatide on insulin resistance and AMPKα-SIRT1 molecular pathway in PCOS rats

**DOI:** 10.1186/s13048-019-0555-8

**Published:** 2019-09-16

**Authors:** Xin Tao, Lisi Cai, Lei Chen, Shuqi Ge, Xuanying Deng

**Affiliations:** 1 Center for Reproductive Medicine, The Third Affiliated Hospital, Sun Yat-sen University, 600, Thianhe Road, Guangzhou, Guangdong People’s Republic of China 510630; 2 Ultrasound Department, The First Affiliated Hospital, Sun Yat-sen University, Guangzhou, Guangdong People’s Republic of China 510080; 3 Center for Republic Medicine, The Sixth Affiliated Hospital of GuangZhou Medical University, The People’s Hospital of Qingyuan, Qingyuan, Guangdong People’s Republic of China 511500

**Keywords:** Polycystic ovary syndrome, Obesity, Insulin resistance, AMPKα, SIRT1

## Abstract

**Aims:**

This study was designed to evaluate the protective effects of AMPKα and SIRT1 on insulin resistance in PCOS rats, and to illuminate the underlying mechanisms.

**Methods:**

An in vitro PCOS model was established by DHEA (6 mg/(100 g•d)), and the rats were randomly divided into the metformin group (MF group, *n* = 11), the exenatide group (EX group, *n* = 11), the PCOS group (*n* = 10), and the normal control group (NC group, *n* = 10). The MF group was administered MF 300 mg/(kg•d) daily. The EX group was subcutaneously injected EX 10μg/(kg•d) daily. After 4 weeks of continuous administration, fasting blood glucose and serum androgen, luteinizing hormone and other biochemical indicators were measured. Western and Real-time PCR were used to determine the expression of AMPKα and SIRT1 in the ovaries of each group.

**Results:**

After 4 weeks of drug intervention, compared with untreated PCOS group, EX group and MF group had visibly decreased body weight (222.64 ± 16.57, 218.63 ± 13.18 vs 238.30 ± 12.26 g, *P* = 0.026), fasting blood glucose (7.71 ± 0.72, 8.17 ± 0.54 vs 8.68 ± 0.47 mmol/L, *P* < 0.01), HOMA-IR (8.26 ± 2.50, 7.44 ± 1.23 vs 12.66 ± 1.44, *P* < 0.01) and serum androgen (0.09 ± 0.03, 0.09 ± 0.03 vs 0.53 ± 0.41 ng/ml, *P* < 0.01) and the expressions of AMPKα and SIRT11 were increased progressively (*P* < 0.05).

**Conclusions:**

Both metformin and exenatide can improve the reproductive and endocrine functions of rats with PCOS via the AMPKα-SIRT1 pathway, which may be the molecular mechanism for IR in PCOS and could possibly serve as a therapeutic target.

## Introduction

The polycystic ovary syndrome (PCOS) is a common endocrine disorder in women of reproductive age with a prevalence of 5–10% [1]. The syndrome is characterized by hyperandrogenism, ovulatory dysfunction and polycystic ovaries. PCOS, a syndrome of unknown etiology, is furthermore associated with accumulation of abdominal fat, obesity (BMI > 30 kg/m^2^) and insulin resistance (IR), which are present in 70–80% of women of PCOS [2]. There is increasing global data linking PCOS to metabolic complications, such as impaired glucose tolerance (IGT), type 2 diabetes (DM2), dyslipidemia, elevated cardiovascular risk factors [3]. Due to the high incidence of obesity and IR in PCOS patients, weight reduction and lifestyle modification have become an important component in the treatment of the disease. However, many patients fail to lose weight or quickly regain fat. Effective intervention is urgently needed to minimize metabolic complications in patients with PCOS.

Insulin sensitizers, especially metformin (MF), have been shown as a pharmaceutical option aiming at not only IR, but also several other aspects of PCOS, including reproductive dysfunctions [4]. In 1994, Velazquez reported for the first time that MF had beneficial effects on reproductive as well as metabolic abnormalities in women with PCOS [5]. Since then, a lot of studies have confirmed the protective impact of MF on IR and obesity in women with PCOS. MF lowers blood glucose and enhances insulin sensitivity by reducing hepatic gluconeogenesis via activating AMP-activated protein kinase (AMPK) pathway [6]. A major limitation of its use is its side effects, which are predominantly gastrointestinal reactions consisting of nausea, diarrhea and bloating. Moreover, The weight loss effect of MF on the basis of lifestyle therapy does not seem to be very satisfactory [7].

Glucagon-like peptide 1 (GLP-1) is an incretin hormone that was primarily described in the 1980s as a proglucagon cleavage products, produced by intestinal cells in response to food intake [8]. It lowers postprandial glucose levels by promoting glucose-dependent insulin secretion, inhibiting glucagon secretion, decelerating the emptying of gastric contents and improving pancreatic β-cell function [9]. However, it is easily degraded by dipeptidyl peptidase IV (DPP-IV), with a half-life less than 2 min, which greatly limits its clinical application. This problem was overcome by the development of synthetic GLP-1 receptor agonists, such as exenatide (EX), which have been clinically used for the treatment of DM2, providing better glycemic control. In an open-label prospective randomized research, 12 weeks of EX treatmet produced a significant weight loss and improved insulin resistance in overweight/obese women with PCOS compared with MF treatment [10]. This is in line with another study, which showed that EX appeared to be superior to MF in restoring menstrual cycles and regulating metabolic disorders [11]. However, its mechanism of improving IR has not yet been addressed in women with PCOS.

In our previous study [12, 13], insulin resistance in PCOS rats was associated with the AMPKα-SIRT1 pathway. Therefore, in this study, we used MF or EX to intervene PCOS rats to compare their influences on metabolic abnormalities and to investigate whether their protective effects were related to the AMPKα-SIRT1 pathway.

## Materials and methods

### PCOS rats models

The Animal Experimental Center of Sun Yat-sen University Medical College (SCXK (GuangDong) 2011–0029) provided fifty female SD rats (25-day-old). These female rats were all specific-pathogen-free (SPF) grades with an average body weight of 79.79 ± 4.18 g. The rats were randomly divided into two groups: PCOS model group (*n* = 37) and normal control group (*n* = 13). The PCOS group rats were subcutaneously injected for 20 days with dehydroepiandrosterone (DHEA 6 mg/(100 g•d)) (Millipore (252805)) and 0.2 ml injectable soybean oil; while the NC group rats were subcutaneously injected with only 0.2 ml of injectable soybean oil. The rats’ weight were recorded daily. After ten days of injections, the rats in both groups were vaginally swabbed daily and the discharge was observed under the microscope throughout three estrous cycles. After the estrous cycle of the PCOS group disappeared or irregular, the PCOS model were considered to have been successfully established. Eight rats were randomly selected (3 from the control group and 5 from the PCOS group) for the fasting blood glucose, serum testosterone and fasting insulin tests, as well as for histological examination of their ovarian issues to further evaluate the efficiency of model establishment.

**Fig. 1:**
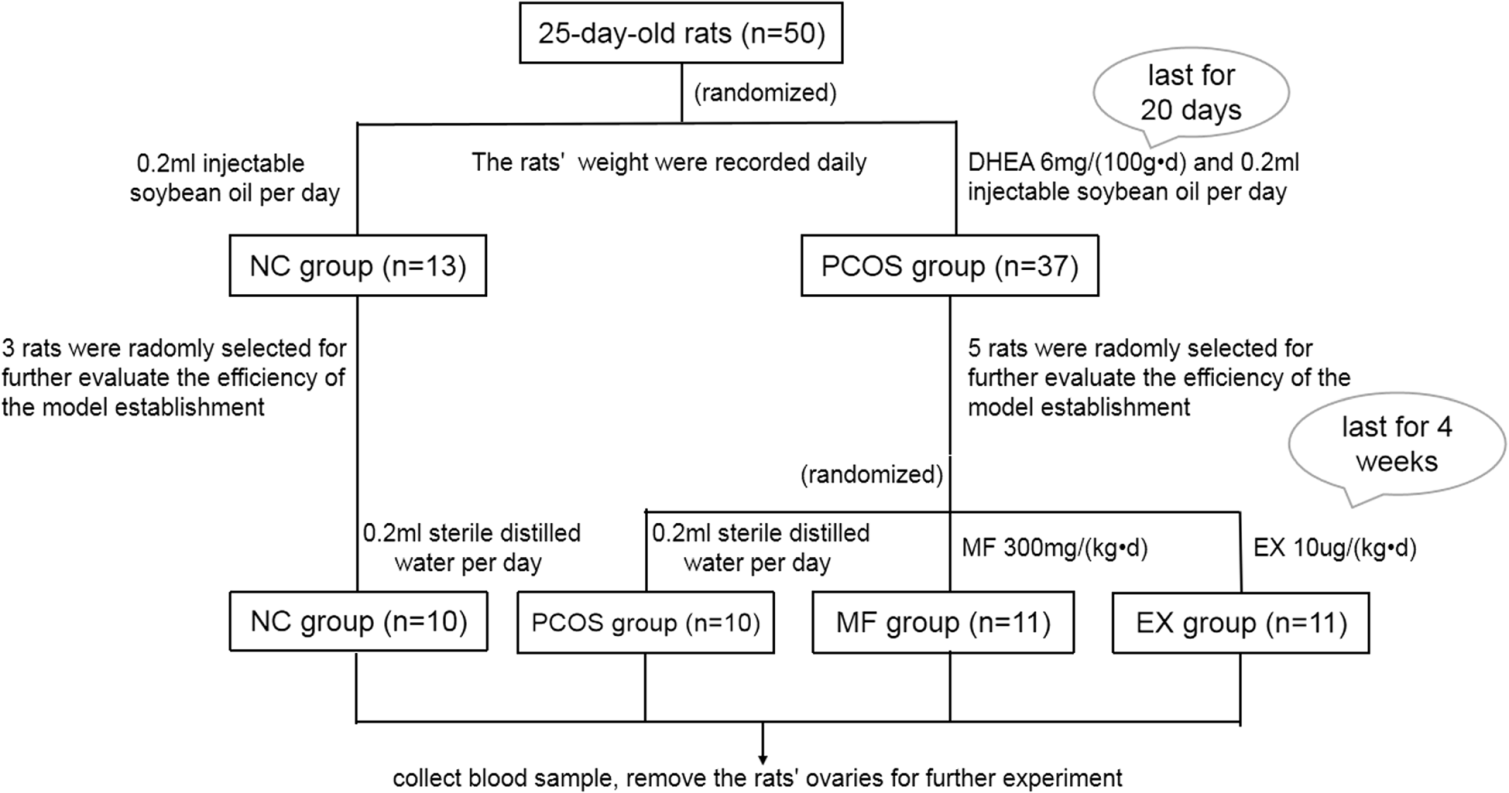
Flow chart of the experiment

The remaining 42 rats were randomly divided into 4 groups: MF group (*n* = 11), EX group (n = 11), PCOS group (*n* = 10) and the NC group (NC group, n = 10). The MF group was administered MF 300 mg/(kg•d) daily, dissolved in 0.2 ml sterile distilled water. The EX group was subcutaneously injected EX 10μg/(kg•d) daily, dissolved in 0.2 ml of sterile distilled water. The PCOS group and the NC group were subcutaneously injected with only 0.2 ml of sterile distilled water every day. All injections lasted for 4 weeks (Fig. 1).

### Blood and ovarian tissue collection

The blood and ovarian tissue collection and the hematoxylin-eosin (HE) staining was done as previously described [12]. In the microscopic examination, 5 fields were randomly selected in every pathological section for observation and the number of immature follicles was counted under high power microscope fields (HPF) (400X). A total of 10 sections were observed [14].

### Western blot assays (WB)

The primary antibody was purchased from Cell Signaling Technology (CST): anti-AMPKα (5832S), anti-pAMPKa1/2 (2535S), anti-SIRT1 (8469S). All corresponding secondary antibodies were purchased from Sino Biological (China, Beijing).

### Quantitative real-time polymerase chain reaction (qPCR)

Total RNA from ovarian tissues was extracted using Trizol reagent (Invitrogen), and cDNA was generated using a reverse transcription kit (Takara (RR047A)). The RT-PCR kit was purchased from Takara (RR820A). Primer sequences were as follows: AMPKα: 5′-TAAACCCACAGAAATCCAAACACC-3′(forward), 5′-ACAACCTTCCATTCATAGTCCAACT-3′(reverse); SIRT1: 5′-AACCACCAAAGCGGAAAAAAAGAA-3′(forward), 5′-CCACAGCAAGGCGAGCATAAATA-3′(reverse); endogenous control β-actin: 5′-CCGTAAAGACCTCTATGCCAACA-3′(forward), 5′-CTAGGAGCCAGGGCAGTAATCTC-3′(reverse).

### Statistical analysis

Data statistics and analysis were performed using SPSS 21.0 software (SPSS Inc., Chicago, IL, USA). The results were expressed in mean ± standard deviation (SD) or median and interquartile ranges. An independent two samples T test was used for homogeneity of variance, otherwise the non-parametric test was used. One-way ANOVA was carried out when multiple comparisons were evaluated. The difference was considered statistically significant at *P* < 0.05.

## Results

### Estrous cycle monitoring and parameters of rats after DHEA pretreatment

Rats in the PCOS group lost their regular estrous cycles and remained in the diestrus phase after DHEA treatment. Whereas the estrous cycle of the control group was still regular at about 4–5 days. As shown in the Table 1A, after 20 days of DHEA treatment, compared with those in the control group, body weights (166.38 ± 7.69 vs 158.92 ± 10.06 g, *P* = 0.008) and fasting blood glucose (FBG) (9.50 ± 0.60 vs 7.90 ± 0.60 mmol/L, *P* = 0.01) in the PCOS group were increased significantly. Fasting insulin levels (FINS) in the PCOS group (30.12 ± 6.63 vs 23.07 ± 2.07 mU/L, *P* = 0.132) were also higher than those in the control group, although the difference was not statistically significant. Furthermore, the PCOS group showed prominent hyperandrogenemia (4.92 ± 2.41 vs 0.12 ± 0.07 ng/ml, *P* = 0.011) and IR (measured by HOMA-IR, 12.63 ± 2.32 vs 8.10 ± 0.93, *P* = 0.02), suggesting the successful establishment of PCOS rats.

**Table 1:** Weight and serum hormone data

Group	Weight(g)	FBG (mmol/L)	FINS (mU/L)	HOMA-IR	T (ng/mL)	LH (mIU/L)
A: Data and Comparison between Control group and PCOS group after continuous injection of DHEA for 20 days						
Control(n = 3)	158.92 ± 10.06	7.90 ± 0.60	23.07 ± 2.07	8.10 ± 0.93	0.12 ± 0.07	3.17 ± 1.08
PCOS(*n* = 5)	166.38 ± 7.69	9.50 ± 0.60	30.12 ± 6.63	12.63 ± 2.32	4.92 ± 2.41	3.64 ± 1.50
*P* value/T test	0.008^*^	0.01^*^	0.132	0.02^*^	0.011^*^	0.661
B: Data between 4 groups after continuous injection of metformin and exenatide for 4 weeks						
NC(n = 10)	222.60 ± 17.88	7.92 ± 0.45	23.38 ± 3.24	8.25 ± 1.36	0.08 (0.06–0.10)	3.02 ± 0.73
PCOS(n = 10)	238.30 ± 12.26^a^	8.68 ± 0.47^a^	32.91 ± 4.27^a^	12.66 ± 1.44^a^	0.35 (0.20–0.99)^a^	3.19 ± 0.85
EX(n = 11)	218.63 ± 13.18	8.17 ± 0.54	20.51 ± 3.53	7.44 ± 1.23	0.08 (0.08–0.10)	2.99 ± 0.57
MF(n = 11)	222.64 ± 16.57	7.71 ± 0.72	25.08 ± 6.44	8.26 ± 2.50	0.09 (0.07–0.12)	2.53 ± 1.00
*P v*alue/one way ANOVA	0.026^*^	< 0.01^*^	< 0.01^*^	< 0.01^*^	< 0.01^*^	0.276

*Mean* Mean value, *SD* Standard Error, *FBG* Fasting blood Glucose, *FINS* Fasting insulin, *T* Testosterone, *HOMA-IR* HOMA insulin Resistance index

*:*P* < 0.05 That means that the difference is statistically significant between groups

^a^: That means the difference between this group and other groups is statistically significant

### Ovarian morphologic changes after DHEA pretreatment

**Fig. 2:**
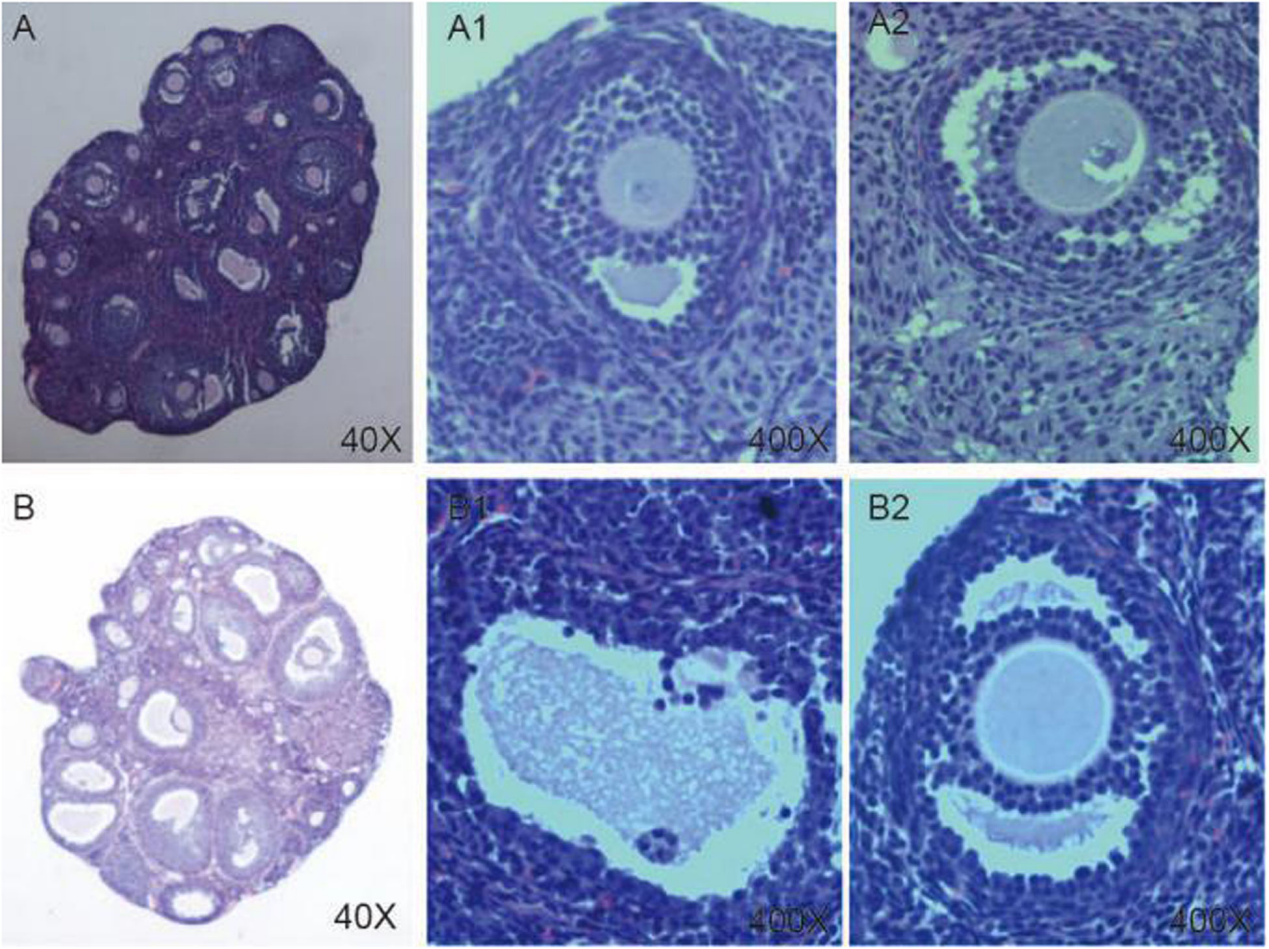
HE staing of the ovaries of rats. **a** The ovaries of the control group rats (40X); A1, A2: Part of figure A (400X). **b** The ovaries of the PCOS group rats (40X); B1, B2: Part of figure B(400X)

In the control group, follicles of different developmental stages and a few corpora lutea were observed. The granulosa cells were orderly arranged in an intact form, mostly in 4–6 layers. However, the number of immature follicles was significantly increased (13.20 ± 2.38 vs 8.00 ± 1.00, *P* = 0.002) in the PCOS group, and the corona radiation of oocytes disappeared, and granulosa cells were arranged loosely in fewer (only 1–3) layers (Fig. 2, Table 2).

**Table 2:** Comparison of the number of immature small follicles between the two groups

Group	The number of immature small follicles /HPF	*P* value/T test
NC	8.00±1.00	0.002*
PCOS	13.20±2.38	0.002*

*HPF* High power field

* *P* < 0.05 That means the difference is statistically significant between groups

### Parameters of rats after metformin or exenatide intervention

As shown in Table 1B, compared with the PCOS group, body weights (222.64 ± 16.57, 218.63 ± 13.18 vs 238.30 ± 12.26 g, *P* = 0.026) and serum testosterone (0.09 ± 0.03, 0.09 ± 0.03 vs 0.53 ± 0.41 ng/ml, *P* < 0.01) in the MF group and EX group were significantly decreased. Moreover, the insulin sensitivity of MF and EX groups had imrpoved (*P* < 0.01). The body weight (218.63 ± 13.18vs 222.64 ± 16.57 g) and HOMA-IR (7.44 ± 1.23 vs 8.26 ± 2.50) of the EX group were lower than those of the MF group, although the difference was not statistically significant. These results demonstrated that MF and EX both can improve metabolic abnormalities in PCOS rats.

### AMPKα and SIRT1 protein and mRNA expression in rat ovaries after metformin or exenatide intervention

**Fig. 3:**
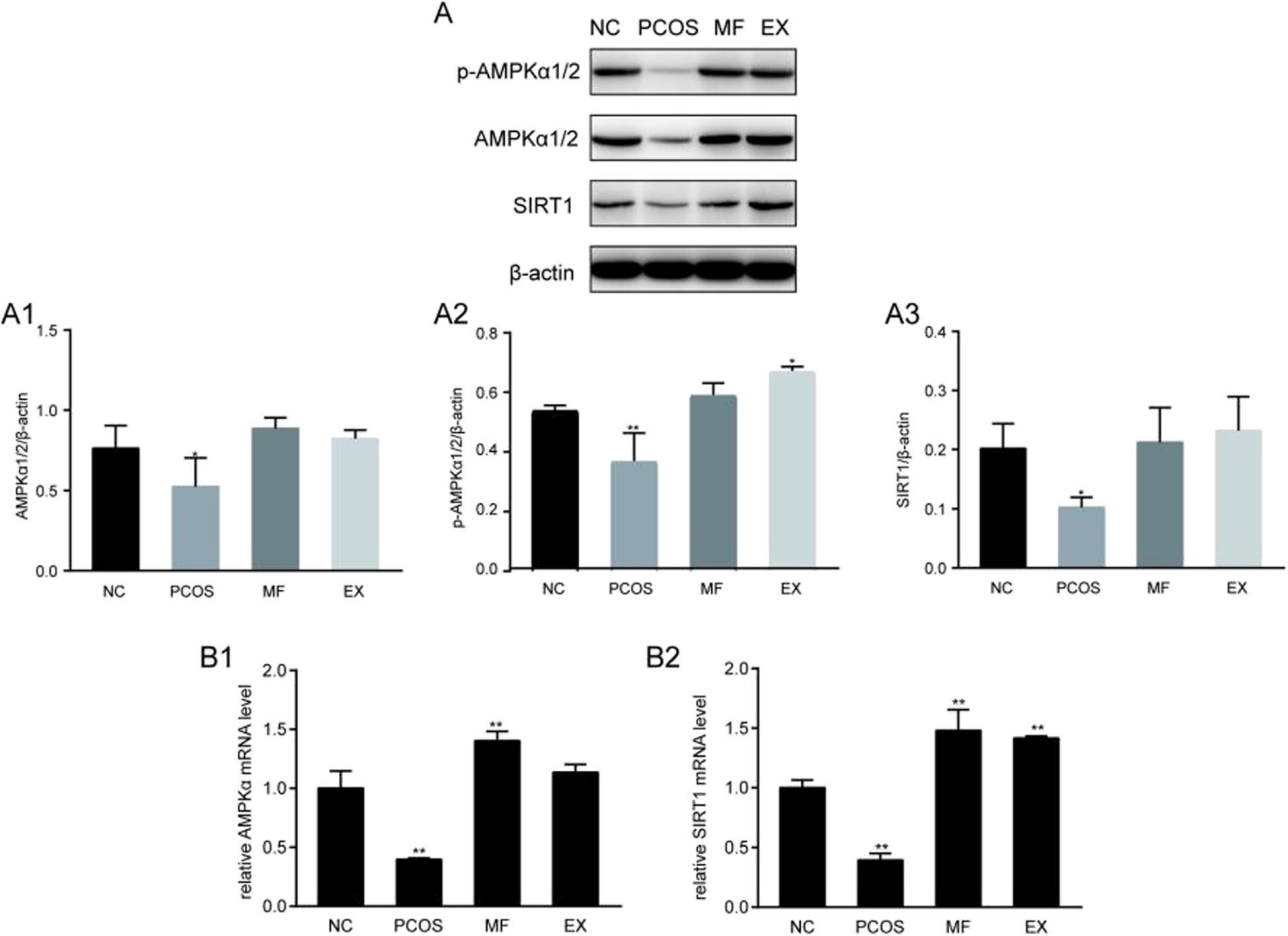
**a** The result of Western blot of the expression of AMPKα and SIRT1 between 4 groups. **b** The result of RT-PCR of the expression of AMPKα and SIRT1 between 4 groups. ^*^P < 0.05, ^**^P < 0.01

DHEA treatment resulted in reduced expression of AMPKα protein, MF or EX treatment increased AMPKα protein expression. The SIRT1 expression was consistent with that of AMPKα in each group (Fig. 3a), suggesting that upregulation of the AMPα-SIRT1 molecular pathway can improve the IR status of PCOS rats.

The expression of AMPKα and SIRT1 mRNA were decreased in the PCOS group, while MF or EX treatment could increased the mRNA expression of AMPKα and SIRT1, and restored the regular menstrual cycle. We conclude from the trend of expression that MF and EX may exert their protective effects on metabolic abnormalities in PCOS rats via AMPKa-SIRT1 pahtway (Fig. 3b).

## Discussion

The clinical manifestation of PCOS is highly heterogeneous. It is a complex reproductive endocrine and psychological disease, which affects the health of women throughout their life [1]. PCOS is related to a series of reproductive, obstetrical, metabolic and psychological symptoms. The clinical manifestations of reproduction and obstetrics include menstrual disorder, hyperandrogenism, sterility and pregnancy concomitant symptoms, such as gestational diabetes mellitus, gestational hypertension, early abortion and neonatal concomitant symptoms [2]. Metabolic clinical manifestations include metabolic syndrome, IGT, type 2 diabetes, cardiovascular diseases and so on. Furthermore, PCOS patients are often accompanied by psychological symptoms, including depression and inferiority, which affect the quality of life [15]. In our study, rats in the PCOS group lost their regular estrous cycle, the microscopic examination revealed the presence of increased number of immature follicles. These results suggested that there were ovulatory disorders and ovarian polycystic changes in PCOS group, which is also an important clinical manifestation of PCOS. In addition, the body weight, serum testosterone and HOMA-IR in PCOS group were significantly higher than those in control group, suggesting that PCOS group was in an apparently IR status and accompanied by obesity, hyperandrogenism. IR seems to be an important determinant of metabolic disorders in patients with PCOS [16]. IR leads to increased insulin secretion from the pancreas to maintain normal blood glucose levels, resulting in compensated hyperinsulinemia, which in turn stimulates fat storage and affects cholesterol and lipoprotein metabolism. Besides, insulin can directly stimulate the activity of cytochrome P450c17α enzyme in follicular membrane and promote the conversion of cholesterol to progesterone and progesterone to androgen. Insulin can also directly promote pituitary secretion of LH, which acts on receptors on theca cells, further increasing androgen production [17]. On the other hand, abdominal obesity and elevated androgen also affect metabolic disorders, which in turn promote the production of insulin resistance. A recent meta analysis [18] used gold standard insulin clamp technique to evaluate the degree of insulin resistance in PCOS. The results showed that the insulin sensitivity of PCOS patients was 27% lower than that of the control group, and this had nothing to do with BMI, age or diagnostic criteria.

MF, an insulin sensitizer, has been introduced as a pharmaceutical option targeting not only IR, but also several other aspects of PCOS [4]. MF counteracts adipose tissue expansion by directly inhibiting lipogenesis. Culturing of pre-adipocytes in the presence of MF resulted in increased phosphorylation of AMPK at Thr172 and the accumulation of significantly less lipid than in non-treated cells [19]. This observation may be related to the potential weight-loss favoring effect of MF. However, MF could not activate purified rat liver AMPK, indicating that it is not a direct activator of AMPK, and its activation of AMPK depends on the presence of intact cells. Shaw et al. [20] showed that the activity of liver AMPK disappeared if liver kinase B1 (LKB1), the AMPK upstream kinase, was knocked out, and MF also lost its hypoglycemic effect. Therefore, MF was thought to act through the LKB1-AMPK pathway.

In addition to its hypoglycaemia action, MF can also protect microvascular endothelial cells from glucose toxicity by a mechanism that may involves SIRT1-mediated growth arrest [21]. AMPKα elevates the expression of SIRT1 by up-regulating the intracellular levels of its co-substrate NAD+ or the activity of nicotinamide [22]. Similarly, SIRT1 can activate AMPK via deacetylation of LKB1, which promotes LKB1 translocation from the nucleus to the cytosol, where it is activated and phosphorylates and activates AMPK [23]. A similar action of MF via the AMPKα-SIRT1 pathway has also been shown in hepatic HepG2 cells under high glucose conditions [24]. This finding is consistent with our study that the levels of AMPKα and SIRT1 in the ovary of PCOS rats were significantly lower than those in the control group. The expressions of AMPKα and SIRT1 were significantly increased after AMPKα agonists treatment, such as MF.

Although MF has been widely used to improve IR in patients with PCOS, many patients can not tolerate its gastrointestinal side effects, and its weight control effect is not satisfactory [7]. In our study, GLP-1 receptor agonists EX and MF significantly improved insulin resistance and endocrine disorder in PCOS, and the average body weight and HOMA-IR of rats in EX group were lower than those in MF group, although the difference was not statistically significant. This may be related to the short duration of intervention. Our results are consistent with the results of a non-blind prospective randomized controlled study [10] of obese PCOS patients. In that study, the experimental group was treated with subcutaneous EX (10 μg bid) for 12 weeks, while the control group was given oral MF (1000 mg bid). The result showed that EX group had more significant weight loss and improved HOMA-IR, and the natural pregnancy rate of EX group was higher than that of MF group. GLP-1, a potent antidiabetic incretin hormone produced by intestinal cells, is widely used for DM2 treatment because of its action to stimulate insulin secretion, suppress glucagon production and release in a glucose-dependent manner. Despite its potent insulinotropic effect, the clinical application of oral GLP-1 is greatly limited by its instability in the gastrointestinal tract, poor absorption efficiency and rapid degradation by DPP4 [25]. Various GLP-1 receptor agonists, such as EX, have been developed to provide prolonged in vivo actions. EX, with a half-life of more than 2.4 h, only increases insulin release in the case of hyperglycaemia and therefore does not cause hypoglycaemia [8]. EX decreases glucagon release after binding to the its receptor (GLP-1R) present on pancreatic endocrine α- and β- cells [26]. GLP-1R is coupled to G protein, which, once activated, increases intracellular cyclic AMP (cAMP) and induces extracellular signal-regulated kinase (ERK) 1/2, protein kinase A (PKA) and phosphoinositol 3 Activation of kinase (PI3K)/protein kinase B (PKB) [27].

Obesity, insulin resistance and hyperandrogenism are often associated with PCOS, improved weight control and glycemic profiles often result in prevention of metabolic syndrome in women with PCOS [16]. In the present study, we found that after 4 weeks of MF or EX treatment, body weight, fasting blood glucose and HOMA-IR were significantly reduced compared with the untreated PCOS group. In addition, after MF or EX treatment, the elevation in AMPKα and SIRT1 expression indicated that AMPKα-SIRT1 pathway might participate in the improvement of metabolic disorder due to MF or EX treatment. Nevertheless, whether the effects of GLP-1 are mediated via the activation of SIRT1 and/or directly via AMPK still requires further studies.

## Conclusion

In conclusion, MF and GLP-1 receptor agonists, such as EX, can significantly improve insulin resistance in PCOS rats, and their action may be in relation to the AMPKα-SIRT1 pathway. Therefore, the AMPKα-SIRT1 pathway is expected to be an important target for the treatment of patients with PCOS. This matter deserves further attention. Larger trials are needed to explore the mechanism of EX in reducing body weight and improving IR in women with PCOS.

## Data Availability

Please contact author for data requests.

## Abbreviations

AMPK: AMP-activated protein kinase
cAMP: cyclic AMP
DHEA: Dehydroepiandrosterone
DM2: Diabetes mellitus 2
DPP-IV: Dipeptidyl peptidase IV
ERK: Extracellular signal-regulated kinase
EX: Exenatide
FBG: Fasting blood glucose
FINS: Fasting insulin
GLP-1: Glucagon-likepeptide1
HE: Hematoxylin-eosin staining
HOMA-IR: Homeostasis model assessment for insulin resistance
HPF: High power field
IGT: Impared glucose tolerance
IR: Insulin resistance
LKB1: Liver kinase B1
NC: Normal control
PCOS: Polycystic ovarian syndrome
PI3K: Phosphoinositol 3 Activation of kinase
PKA: Protein kinase A
qPCR: Real-time Quantitative PCR Detecting System
SD: Standard deviation
SIRT1: Sirtuin1
SPF: Specific-pathogen-free
